# Supercritical CO_2_-Assisted Impregnation of Absorbable Surgical Sutures with Carvacrol and Benzydamine Hydrochloride: Comparative In Vitro Release Profiles and Drug Release Kinetics

**DOI:** 10.3390/polym18141698

**Published:** 2026-07-10

**Authors:** Aysun Akpınar, Merve Öztürk, Önder Aybastıer, Halil Çelik, Gezu Ketema Janka, Hüseyin Aksel Eren, Semiha Eren

**Affiliations:** 1 Department of Periodontology, Faculty of Dentistry, Bursa Uludağ University, Bursa 16120, Türkiye; 2 Department of Textile Engineering, Faculty of Engineering, Bursa Uludağ University, Bursa 16059, Türkiye; 532117001@ogr.uludag.edu.tr (M.Ö.); 53218273@ogr.uludag.edu.tr (G.K.J.); aksel@uludag.edu.tr (H.A.E.); semihaeren@uludag.edu.tr (S.E.); 3 Department of Chemistry, Faculty of Science and Arts, Bursa Uludağ University, Bursa 16210, Türkiye; aybastier@uludag.edu.tr; 4 Department of Oral and Dental Health, Vocational School of Health Care Services, Istinye University, Istanbul 34485, Türkiye; halil.celik@istinye.edu.tr

**Keywords:** impregnation process, drug delivery, carvacrol, benzydamine, dental suture

## Abstract

The development of bioactive surgical sutures capable of delivering therapeutic agents directly at the wound site has gained increasing attention in biomedical research. Functionalized sutures may provide localized antimicrobial or anti-inflammatory activity, potentially reducing postoperative complications and promoting tissue healing. In this study, absorbable surgical sutures were impregnated with carvacrol, a natural phenolic compound with well-known antimicrobial properties, using supercritical carbon dioxide (scCO_2_) technology. The impregnation process was carried out at 35 °C and 10 MPa for 120 min, allowing for the incorporation of carvacrol into the polymeric matrix of the sutures. The in vitro release behavior of the impregnated sutures was evaluated in phosphate-buffered saline (PBS, pH 7.4) at 37 °C over an 8-day period. The concentration of released compounds was determined by UV–Vis spectrophotometry using previously established calibration curves. An analysis of the experimental release data demonstrated that both carvacrol and benzydamine hydrochloride (HCl) (Tantum Verde^®^) exhibited a sustained release profile throughout the incubation period. Carvacrol release increased progressively from 2.02 ± 0.15 ppm on day 1 to 7.45 ± 0.15 ppm on day 7, followed by a slight stabilization on day 8 (7.25 ± 0.31 ppm). Similarly, benzydamine HCl (Tantum Verde^®^) release increased from 1.83 ± 0.11 ppm on day 1 to 3.29 ± 0.13 ppm on day 8. Release kinetics were analyzed using the Korsmeyer–Peppas model, indicating that the release mechanism was predominantly diffusion-controlled during the initial stage of the experiment. The results demonstrate that supercritical CO_2_ impregnation is an effective solvent-free technique for incorporating bioactive compounds into absorbable sutures, enabling controlled release under physiological conditions.

## 1. Introduction

Surgical sutures are essential biomaterials widely used to approximate tissues and promote wound healing following surgical procedures [1]. In addition to their mechanical function, sutures may influence the local biological environment during the healing process [2]. However, the presence of sutures can also facilitate microbial colonization and biofilm formation, potentially leading to postoperative infections and delayed wound healing. For this reason, the development of functionalized sutures capable of delivering bioactive compounds has attracted considerable interest in recent years [3].

Bioactive or drug-loaded sutures represent an effective strategy for localized drug delivery directly at the surgical site. Compared with systemic drug administration, localized delivery systems provide higher drug concentrations at the target area while minimizing systemic side effects [4,5].

In particular, biodegradable or absorbable sutures are advantageous because they gradually degrade in the body after fulfilling their function, eliminating the need for a second surgical procedure for removal. Incorporating therapeutic agents into absorbable sutures may therefore provide sustained antimicrobial or anti-inflammatory activity during the critical early stages of wound healing [4,6].

Various techniques have been proposed for loading bioactive compounds into polymeric biomaterials, including solvent casting, coating, and immersion methods [7]. However, conventional methods often require organic solvents and may result in uneven drug distribution or the limited penetration of the active compound into the polymer matrix [8]. In this context, supercritical fluid technology has emerged as a promising alternative for the impregnation of polymers with therapeutic agents. Supercritical carbon dioxide (scCO_2_) is particularly attractive due to its low critical temperature, non-toxicity, and ability to diffuse efficiently into polymeric structures [8,9,10]. During the supercritical impregnation process, the active compound dissolves in the supercritical fluid and penetrates the polymer matrix, allowing for the deeper and more homogeneous incorporation of the compound without the use of harmful solvents [11].

Among natural bioactive compounds, carvacrol, a phenolic monoterpenoid commonly found in oregano and thyme essential oils, has gained attention due to its strong antimicrobial, antioxidant, and anti-inflammatory properties [12,13]. Previous studies have demonstrated that carvacrol exhibits significant inhibitory effects against a wide range of pathogenic microorganisms, including oral and wound-associated bacteria [13,14,15]. These properties make carvacrol a promising candidate for incorporation into biomedical materials aimed at preventing infection and supporting tissue healing [14].

Despite the increasing interest in functionalized biomaterials, studies investigating the incorporation of carvacrol into absorbable suture materials using supercritical impregnation technology remain limited [14,16]. Understanding the release behavior of such systems is crucial for evaluating their potential as localized drug delivery platforms.

Therefore, the aim of the present study was to impregnate absorbable surgical sutures with carvacrol using supercritical CO_2_ technology and to investigate the in vitro release profile of the incorporated compound in physiological conditions. The results of this study may contribute to the development of novel bioactive sutures capable of providing the controlled release of antimicrobial agents during the early stages of wound healing.

## 2. Materials and Methods

Absorbable polyglycolic acid (PGA) surgical sutures (Pegesorb^®^ 4/0, Doğsan Medical, Trabzon, Türkiye) were used as the polymeric biomaterial for the supercritical impregnation process. Carvacrol (IC Bitkisel Guner Ceylan 95%, Türkiye), a phenolic monoterpenoid with known antimicrobial properties, was used as the active compound. Benzydamine HCl (Tantum Verde^®^, Angelini Pharma, Istanbul, Türkiye) was also impregnated as a comparative compound under identical experimental conditions. Carbon dioxide (CO_2_) was used as the supercritical fluid during the impregnation process.

Phosphate-buffered saline (PBS, pH 7.4) was prepared and used as the release medium in the in vitro release experiments. All reagents used in the preparation of the buffer solution were of analytical grade.

### 2.1. Supercritical CO_2_ Impregnation of Sutures

**Figure 1:**
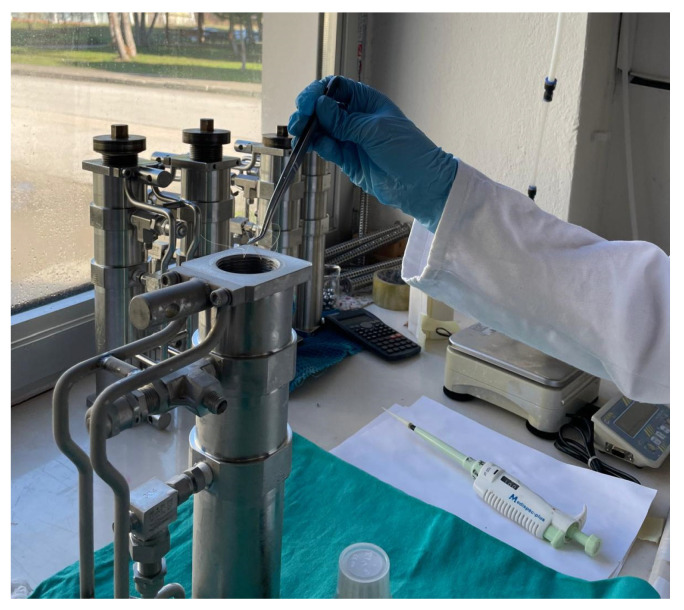
The supercritical carbon dioxide (scCO_2_) impregnation system used for the incorporation of carvacrol and benzydamine HCl into absorbable surgical sutures.

The incorporation of bioactive compounds into the absorbable sutures was carried out using a supercritical carbon dioxide (scCO_2_) impregnation technique. The sutures were exposed to either carvacrol or benzydamine HCl under controlled temperature and pressure conditions in a high-pressure supercritical system (Figure 1). Three independently prepared and independently impregnated PGA suture specimens were used for each compound. Each specimen was incubated separately in PBS under identical experimental conditions, and the concentration released from each specimen was determined individually. The values reported in Table 1 represent independent experimental replicates (*n* = 3), and the data are expressed as the mean ± standard deviation.

scCO_2_ experiments were performed using a Rapid Xiamen Model H12 oil bath system (DyeCOO, VDL KTI, Nijverheidsstraat, Mol, Belgium). In the first stage, the sutures were placed into stainless steel tubes with an internal volume of 290 mL. Subsequently, a predetermined amount of carvacrol or benzydamine HCl was added into the tubes, and the tube lids were tightly closed.

The sealed tubes were then placed in a deep freezer for at least 15 min to facilitate the filling of carbon dioxide gas into the tubes. The density (d) of CO_2_ gas was determined using the NIST Chemistry WebBook, based on the temperature and pressure conditions applied in the experiment. The amount of CO_2_ gas (g) was then calculated using the formula d = m/V. Following CO_2_ loading, the tubes were placed in a supercritical system, and the impregnation process was initiated [17,18].

Impregnation was performed at a temperature of 35 °C and a pressure of 10 MPa for 120 min [19]. Under these conditions, CO_2_ reached the supercritical state and acted as a solvent, facilitating the diffusion of the active compounds into the polymeric structure of the sutures. After the impregnation period, the system was gradually depressurized, and the impregnated sutures were removed and stored under appropriate conditions prior to further analyses.

For comparison purposes, sutures were impregnated under identical supercritical carbon dioxide conditions without the addition of carvacrol or benzydamine HCl. Additionally, sutures were immersed in a solution containing carvacrol or benzydamine HCl at room temperature for 2 h.

The mass of the impregnated sutures was recorded before the release experiments. The carvacrol-loaded suture had a mass of 8.5 mg, while the benzydamine HCl-loaded suture had a mass of 8.8 mg.

### 2.2. In Vitro Release Study

The release behavior of the impregnated compounds was evaluated through an in vitro release study conducted under physiological conditions.

Each suture sample was placed in a sealed glass vial containing 10 mL of PBS solution (pH 7.4). The samples were incubated at 37 °C in a laboratory incubator to simulate physiological temperature. The release experiments were conducted for a total duration of 8 days.

At predetermined time points (days 1, 2, 3, 5, 6, 7, and 8), spectrophotometric measurements of the solution were performed to determine the concentration of the released compounds. Absorbance values at 273 nm for carvacrol and 301 nm for benzydamine HCl were used. Calibration curves prepared for carvacrol and benzydamine HCl (Tantum Verde^®^) were used to calculate the concentration of the released substances in the solution.

Three independently prepared and independently supercritical CO_2_-impregnated PGA suture specimens were used for each compound (*n* = 3). Each specimen was incubated separately in PBS under identical experimental conditions, and the released compound was quantified individually by UV–Vis spectrophotometry. The values reported in Table 1 (Replicates 1–3) represent independent experimental replicates and are expressed as the mean ± standard deviation (SD).

### 2.3. Spectrophotometric Analysis

The concentration of carvacrol and benzydamine HCl released into the PBS medium was determined using a UV–Vis spectrophotometer (UV-6300PC, VWR, Radnor, PA, USA). Spectrum scans (200–800 nm) were performed during the incubation period, and the concentrations of the released compounds were calculated based on previously established calibration curves. The results were expressed in parts per million (ppm).

### 2.4. Tensile Strength Measurement of Absorbable Surgical Sutures

**Figure 2:**
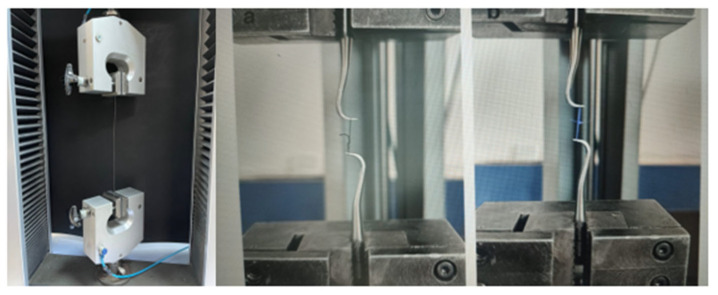
Tensile strength evaluation of untreated, supercritical CO_2_-treated, conventionally impregnated, and carvacrol- or benzydamine HCl-loaded absorbable surgical sutures.

The tensile strength of carvacrol- or benzydamine HCl-impregnated absorbable surgical sutures under scCO_2_ was evaluated using a universal testing machine (AG-X 5 kN, Shimadzu Corporation, Kyoto, Japan) in accordance with EN ISO 2062:2009 (Geneva, Switzerland) (Figure 2). The instrument was equipped with a 5 kN load cell. The tensile strength of untreated, scCO_2_-treated, and conventionally impregnated samples was also evaluated to investigate the effect of loaded agents comprehensively. All measurements were tested in triplicate at a crosshead speed of 10 mm/min with a gauge length of 100 mm until failure. Statistical analysis was performed using Microsoft Excel. The results are expressed as the mean ± standard deviation (SD). A one-way analysis of variance (ANOVA) was conducted to evaluate differences among groups, with *p* < 0.05 considered statistically significant.

## 3. Results

The in vitro release profiles of carvacrol and benzydamine HCl from the supercritical CO_2_-impregnated absorbable sutures were evaluated in PBS (pH 7.4) at 37 °C over an 8-day period. The concentrations of the released compounds in the medium were determined using UV–Vis spectrophotometry and expressed in ppm.

The release profiles of both carvacrol and benzydamine HCl differed between the soaking and supercritical CO_2_ impregnation methods. In the soaking group, carvacrol concentrations increased from 1.53 ppm on day 1 to 2.58 ppm on day 7, followed by a slight decrease to 2.54 ppm on day 8. Benzydamine HCl concentrations in the soaking group increased from 1.40 ppm on day 1 to 1.96 ppm on day 8.

In the supercritical CO_2_ group, higher release values were observed throughout the experimental period. Carvacrol concentrations increased from 2.02 ppm on day 1 to 7.45 ppm on day 7, with a slight decrease to 7.25 ppm on day 8. Benzydamine HCl concentrations increased from 1.83 ppm on day 1 to 3.29 ppm on day 8. Overall, the supercritical impregnation group exhibited greater release values for both active compounds compared to the soaking group during the entire release period.

Overall, both compounds exhibited a progressive release pattern during the initial days of incubation, suggesting diffusion-driven release from the polymer matrix. The release rate slowed after the first few days, indicating a tendency toward stabilization as equilibrium between the polymer matrix and the surrounding medium was approached.

The release behavior observed in this study indicates that the supercritical impregnation process successfully incorporated the bioactive compounds into the suture material and enabled their sustained release under physiological conditions.

To better understand the mechanism governing the release of bioactive compounds from the impregnated sutures, the experimental data were evaluated using the Korsmeyer–Peppas model [20], which is commonly applied to describe drug release from polymeric delivery systems.

The model is expressed by the following equation:

log(M_t_/M_∞_) = log k + n log t

For cylindrical or filamentous polymeric systems such as sutures, an n value below 0.45 indicates Fickian diffusion [21], values between 0.45 and 0.89 suggest anomalous transport, and values close to 0.89 correspond to case II transport dominated by polymer relaxation or degradation [22,23].

An analysis of the experimental release data indicated that the release of carvacrol from the impregnated suture followed a diffusion-controlled mechanism during the initial days of incubation. The gradual increase in concentration observed between days 1 and 3, followed by a stabilization phase, suggests that the release process was mainly governed by the diffusion of the compound from the polymer matrix into the surrounding PBS medium.

Similarly, the benzydamine HCl-loaded suture exhibited a release pattern characterized by a rapid initial increase in concentration followed by a slower release phase (Table 2). This behavior may be attributed to the presence of a fraction of the compound located near the surface of the polymer, resulting in an initial burst release, followed by the diffusion-driven release of the compound embedded within the polymeric structure.

Overall, the release profiles of both compounds indicate that the supercritical CO_2_ impregnation process enabled the incorporation of bioactive molecules within the polymer matrix, allowing for the controlled diffusion of the compounds into the surrounding medium. The observed release behavior is consistent with previously reported drug delivery systems based on biodegradable polymeric matrices [24,25].

**Figure 3:**
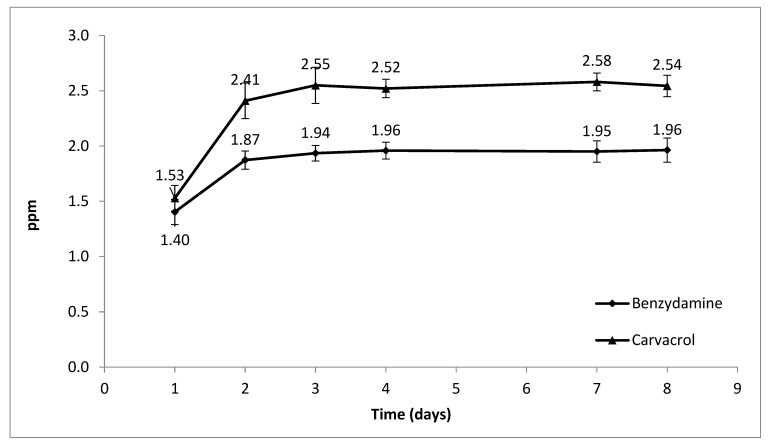
Release profiles of carvacrol and benzydamine HCl from suture materials prepared using soaking impregnation method under physiological conditions over 8 days. Data are presented as mean ± standard deviation (ppm).

**Figure 4:**
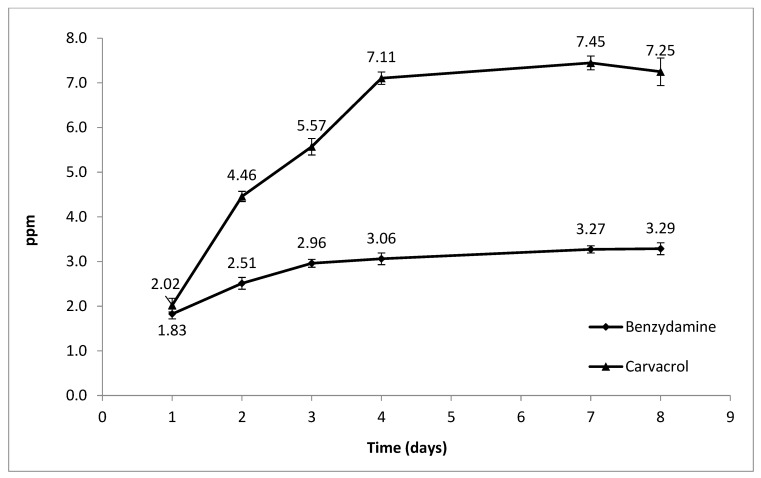
Release profiles of carvacrol and benzydamine HCl from suture materials prepared using supercritical CO_2_ impregnation method under physiological conditions over 8 days. Data are presented as mean ± standard deviation (ppm).

**Figure 5:**
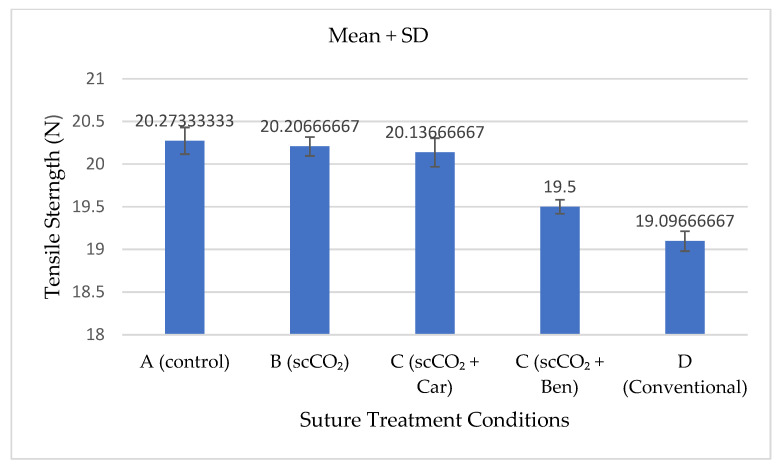
Tensile strength of surgical sutures under different treatments.

In addition to release performance, the effects of impregnation processes on the tensile strength properties of absorbable surgical sutures were also investigated. Therefore, the strength of untreated, conventionally impregnated, supercritical CO_2_-treated and carvacrol- or benzydamine HCl-loaded sutures under supercritical CO_2_ was evaluated, and the results are provided in Figure 3 and Figure 4. The results show that supercritical CO_2_ treatment alone does not affect the tensile strength of the surgical sutures (Figure 5). However, the sutures loaded with carvacrol and benzydamine HCl showed a slight decrease in tensile strength, with benzydamine HCl showing a pronounced reduction in tensile strength compared to carvacrol. The greatest decrease in tensile strength was observed with the sutures loaded with carvacrol or benzydamine HCl using a conventional method, suggesting that the scCO_2_ impregnation process better preserves the structural integrity of the polymer matrix.

Furthermore, the results were analyzed by a one-way analysis of variance (ANOVA), and the results are summarized in Table 3. The analysis detected a significant difference in tensile strength among the groups (F = 47.388, *p* < 0.001), with between-group variability being much higher than within-group variability (MS between = 0.8088; MS within = 0.0171). These findings confirm that the applied treatments have a significant effect on measured tensile strength, and the low within-group variance demonstrated that the experimental results exhibit good consistency and reproducibility.

These findings suggest that supercritical impregnation technology can be used to develop functionalized absorbable sutures capable of providing the sustained local delivery of antimicrobial or anti-inflammatory agents during the early stages of wound healing.

## 4. Discussion

In the present study, carvacrol release from the impregnated suture was characterized by a gradual increase during the early incubation period, followed by a tendency toward stabilization. This profile is consistent with a predominantly diffusion-controlled mechanism, suggesting that carvacrol was incorporated within the polymer matrix and released progressively into the surrounding PBS medium [26]. Such behavior agrees with previous reports showing that bioactive agents embedded in biodegradable polymeric carriers often exhibit an initial diffusion-governed release phase, particularly when the matrix remains structurally intact and erosion is not yet the dominant process [27,28].

By contrast, benzydamine HCl displayed a more pronounced early release followed by a slower release stage, consistent with a biphasic pattern frequently described in polymer-based drug delivery systems [29]. This type of response is commonly attributed to an initial burst release of molecules located near or at the surface of the carrier, followed by the sustained diffusion of the fraction retained deeper within the polymer network. From a therapeutic standpoint, such a profile may be advantageous, as the early burst can provide a rapid local pharmacological effect, while the subsequent slower phase may help maintain the drug concentration during the initial wound healing period [30,31]. Similar burst-plus-sustained profiles have been described in drug-eluting suture systems developed for local anesthesia, analgesia, and anti-inflammatory delivery [5].

The overall behavior observed for both compounds also supports the suitability of supercritical CO_2_ impregnation as a loading strategy for temperature-sensitive bioactives [32,33]. Supercritical impregnation is particularly attractive because it enables the incorporation of active molecules into polymeric devices under relatively mild thermal conditions and without the use of conventional organic solvent residues [34]. A recent study [35] on bioresorbable sutures loaded by supercritical CO_2_, including ketoprofen-impregnated suture threads and PLA-based suture materials loaded with anti-inflammatory drugs, similarly emphasized the ability of this technique to promote drug diffusion into the polymer and generate sustained local release profiles.

Due to its low viscosity and high diffusivity, when supercritical carbon dioxide (scCO_2_) penetrates biodegradable polymer matrices (e.g., PLGA or silk-based structures), it increases the free volume between polymer chains, thereby inducing thermodynamic swelling. This plasticizing effect of scCO_2_ acts as a fundamental ‘working mechanism’ by temporarily depressing the polymer’s glass transition temperature (Tg), which facilitates the transport of small-molecule anti-inflammatory and analgesic agents, such as benzydamine HCl, deep into the polymer matrix. Upon the depressurization of the system, as scCO_2_ rapidly transitions to the gas phase and escapes the structure, the polymer matrix reverts to its original conformation, homogeneously entrapping the benzydamine HCl molecules within its network. This temporary structural modification, as verified by FTIR spectroscopy and mechanical tensile testing, preserves the physicochemical integrity of the polymer without leaving behind toxic organic solvent residues, thereby enabling controlled drug release profiles at the targeted site (e.g., periodontal pockets) [36].

More broadly, the present findings are in line with the growing literature on drug-eluting and biologically active sutures [36], which highlights their dual role as wound closure devices and localized therapeutic platforms. Reviews on functional sutures emphasize that controlled local delivery may reduce surgical site complications, improve wound management, and decrease the need for additional systemic or topical medication [37,38]. In this context, the distinct release behaviors of carvacrol and benzydamine HCl observed here may be clinically relevant: the more gradual release of carvacrol may support prolonged local antimicrobial action, whereas the faster initial release of benzydamine HCl may be better suited for early anti-inflammatory or analgesic support.

In periodontology, sutures are routinely used in a wide range of procedures, including flap surgery, crown lengthening, mucogingival interventions, graft stabilization, and regenerative approaches, where the maintenance of primary wound closure and flap stability is essential for predictable healing outcomes [39,40]. In the oral environment, however, the suture line is continuously exposed to saliva, mechanical stress, and a dense microbial load, making sutures not only mechanical wound closure devices but also attractive candidates for localized therapeutic delivery. Within this context, the distinct release kinetics of carvacrol and benzydamine HCl observed in the present study may imply different periodontal indications. Carvacrol, which demonstrated a more gradual and sustained release profile, may be particularly advantageous in periodontal or peri-implant surgical sites where prolonged local antimicrobial and anti-inflammatory support is desirable, especially during the early healing phase in tissues at risk of plaque accumulation or bacterial recolonization. Experimental and review data have highlighted the anti-inflammatory, antioxidant, and periodontal protective potential of carvacrol, supporting its possible use in sites where longer-lasting bioactivity may be beneficial [13,15]. By contrast, benzydamine HCl, which showed a more pronounced initial release followed by a slower phase, may be better suited for indications in which rapid early analgesic and anti-inflammatory action is clinically preferable, such as immediately after crown lengthening, periodontal flap surgery, or other soft-tissue procedures associated with postoperative pain and discomfort [41]. In clinical use, benzydamine HCl is applied topically at formulation-dependent therapeutic doses. Reported adult posology includes 0.15–0.3% oromucosal sprays corresponding to approximately 2.04–12.24 mg/day, 3 mg lozenges administered three times daily, and 15 mL of 1.5 mg/mL mouthwash used three times daily [42]. In this context, the sustained release of benzydamine HCl observed from the impregnated sutures may be interpreted as a localized delivery approach rather than a systemic dosing strategy. Although the released concentrations were lower than conventional topical exposure levels, the direct placement of the suture at the surgical site may allow benzydamine HCl to act locally during the early postoperative period. This interpretation is consistent with the established local anti-inflammatory and analgesic effects of benzydamine HCl in oral care and periodontal postoperative management.

A limitation of the present study is the absence of the direct antimicrobial validation of the released compounds. Although carvacrol is widely recognized for its antimicrobial properties, the antibacterial activity of the concentrations released from the impregnated sutures under physiological conditions was not directly investigated in this study. Therefore, it remains unclear whether the released concentrations are sufficient to inhibit the growth of clinically relevant oral pathogens. Future studies should evaluate the antimicrobial efficacy of these released compounds against oral microorganisms in order to better determine their potential clinical applicability and therapeutic effectiveness.

## 5. Conclusions

Therefore, beyond confirming the feasibility of supercritical CO_2_ impregnation, the present findings suggest that compound-specific release kinetics may allow absorbable sutures to be tailored according to the clinical priorities of different periodontal indications, with benzydamine HCl favoring early symptomatic relief and carvacrol favoring more sustained local antimicrobial and biologically supportive action.

## Figures and Tables

**Table 1:** The release profile of carvacrol and benzydamine HCl from supercritical CO_2_-impregnated suture materials under physiological conditions over an 8-day period. The absorbance values measured at 273 nm for carvacrol and 301 nm for benzydamine HCl were converted to concentration values (ppm) using the corresponding calibration curves.

	Day	Replicate 1 (Abs)	Replicate 2 (Abs)	Replicate 3 (Abs)	Replicate 1 (ppm)	Replicate 2 (ppm)	Replicate 3 (ppm)	Mean (ppm)	SD
**Carvacrol**	1	0.0283	0.0262	0.0301	2.03	1.87	2.17	2.02	0.15
	2	0.0580	0.0609	0.0592	4.35	4.58	4.45	4.46	0.11
	3	0.0758	0.0739	0.0711	5.74	5.59	5.38	5.57	0.18
	4	0.0915	0.0950	0.0932	6.97	7.25	7.10	7.11	0.14
	7	0.0995	0.0956	0.0978	7.59	7.29	7.46	7.45	0.15
	8	0.0996	0.0922	0.0935	7.60	7.02	7.13	7.25	0.31
**Benzydamine HCl**	1	0.0548	0.0521	0.0492	1.93	1.83	1.72	1.83	0.11
	2	0.0728	0.0661	0.0701	2.63	2.37	2.53	2.51	0.13
	3	0.0833	0.0815	0.0788	3.04	2.97	2.87	2.96	0.09
	4	0.0872	0.0805	0.0836	3.19	2.93	3.05	3.06	0.13
	7	0.0889	0.0914	0.0873	3.26	3.36	3.20	3.27	0.08
	8	0.0935	0.0872	0.0881	3.44	3.19	3.23	3.29	0.13

**Table 2:** Korsmeyer–Peppas kinetic parameters for the release of carvacrol and benzydamine HCl from supercritical CO_2_-impregnated suture materials under physiological conditions.

Compound	N	k	R^2^	Release Mechanism
Carvacrol	0.594	0.341	0.860	Anomalous transport
Benzydamine HCl	0.272	0.607	0.896	Fickian diffusion

**Table 3:** One-way ANOVA results of tensile strength measurements.

Source of Variation	SS	df	MS	F	*p*-Value	F Crit
Between Groups	3.23502667	4	0.80875667	47.3880859	1.8169 × 10^−6^	3.47804969
Within Groups	0.17066667	10	0.01706667			
Total	3.40569333	14				

## Data Availability

The original contributions presented in this study are included in the article. Further inquiries can be directed to the corresponding author.
